# Applied Trends in Magnetic Rare Earth/Transition Metal Alloys and Multilayers

**DOI:** 10.3390/s21165615

**Published:** 2021-08-20

**Authors:** Juan Antonio González, Juan Pedro Andrés, Ricardo López Antón

**Affiliations:** 1 Instituto Regional de Investigación Científica Aplicada (IRICA), Universidad de Castilla-La Mancha, 13071 Ciudad Real, Spain; j.a.gonzalez@uclm.es (J.A.G.); juanpedro.andres@uclm.es (J.P.A.); 2 Departamento de Física Aplicada, Universidad de Castilla-La Mancha, 13071 Ciudad Real, Spain

**Keywords:** spintronics, multilayers, ferrimagnetism, spin valves, spin–orbit torque, information storage, all-optical switching

## Abstract

Ferrimagnetic thin films formerly played a very important role in the development of information storage technology. Now they are again at the forefront of the rising field of spintronics. From new, more efficient magnetic recording media and sensors based on spin valves to the promising technologies envisaged by all-optical switching, ferrimagnets offer singular properties that deserve to be studies both from the point of view of fundamental physics and for applications. In this review, we will focus on ferrimagnetic thin films based on the combination of rare earths (RE) and transition metals (TM).

## 1. Introduction

**Figure 1:**
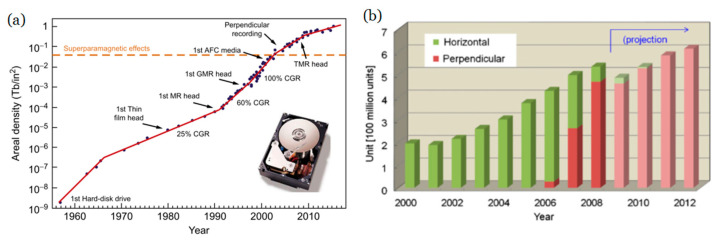
(**a**) Time evolution of the areal bit density since the first commercial magnetic hard drive disk became available in the late 1950s. The inset shows a top view of the IMB hard drive Ultrastar 36ZX with the cover removed. This model was developed in 1999 using GMR head technology and reached an areal density of 3.5 Gb/in^2^. Reprinted from [1] with permission from Elsevier. (**b**) Shift to perpendicular magnetic recording in hard disks. Reprinted with permission from ref. [6] 2012 Elsevier.

The technological advance that has taken place since the middle of the 20th century cannot be understood without taking into account the role played by different systems of storage information. Since the birth of computers, the density of storage has increased exponentially (see Figure 1) and magnetic materials, especially those in thin film form, have been at the basis of much technological development. One of the reasons that magnetism is at the center of these technologies is that it allows action without physical contact, simplifying and making the reading/writing process much quicker. At the same time, the miniaturization of electronic circuits has drastically reduced the size of computers.

Nearly 90% of information storage technologies are based on magnetic materials in thin film or multilayered (ML) form [1]. Each bit of information is recorded in a small part of the material, which has its magnetic moment aimed towards a certain direction or to the opposite, hence representing the two possible states of a binary code. The development of storage technologies, together with magnetic sensors and actuators, was fueled thanks to two essential discoveries: interlayer coupling [2] and giant magnetoresistance (GMR) [3]. In the first case, the coupling between two magnetic layers within an heterostructure depends both on the sign (parallel or antiparallel) and on the thickness of the layer between them, as the interaction shows an oscillatory behavior. In the second case (GMR), the resistance of a multilayer made of ferromagnetic layers separated by a non-magnetic spacer strongly changes when the parallel/antiparallel arrangement of layers is changed by the application of an external field. The ordering of the moments in the layers changes from a natural high-resistance antiparallel arrangement at zero field to a less resistive parallel state when a moderate field is externally applied. This phenomenon is enough to fabricate a sensor (called a spin valve) that can detect the magnetic state of a bit in a small region of a hard disk, thereby giving access to the information previously written. Figure 1 shows the constant increase in the storage density during the last 50 years, and the changes in slope that different technologies have allowed for. Notice that the vertical scale is logarithmic as the size of the bits decreases around nine orders of magnitude in this period of time. It is noteworthy that spin valves can also be used in other kinds of sensors, e.g., industrial applications (integrated magnetic compass, angular and linear sensors) [4] and biomedical applications (integrated cell cytometers, microelectrodes for neuronal magnetic field probing) [5].

**Figure 2:**
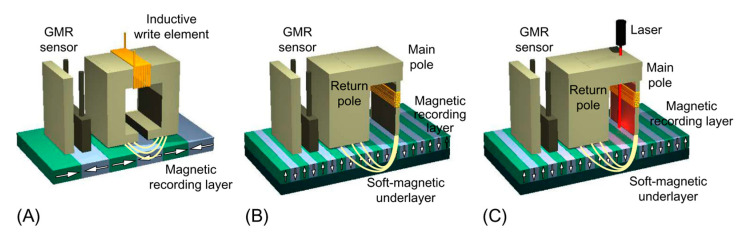
Sketches comparing (**A**) longitudinal magnetic recording (LMR), (**B**) perpendicular magnetic recording (PMR), and (**C**) heat-assisted magnetic recording (HAMR) technologies. In LMR and PMR, the data bits are aligned parallel and perpendicular to the surface of the disk, respectively. The latter configuration reduces the repelling forces between bits and enables higher write magnetic fields, allowing higher areal recording densities. HAMR uses thermal laser heating of the magnetic medium to write data at high temperatures, enabling the use of smaller write magnetic fields and thus magnetic recording layers with smaller grain size and higher anisotropy. This not only improves the long-term stability of the magnetic information but also allows to further increase the areal bit density Reprinted with permission from ref. [1] 2018 Elsevier.

An important limitation showed up when the areal density of information approached 0.5 Tb/in^2^: the so-called superparamagnetic limit [7]. It is of key importance that, once written, the bits can keep their orientation over a long period of time so that we can have access to the information for a long time. Provided that the magnetic anisotropy depends on the product of a constant and the volume of the material (K_u_·V) [8], making smaller bits reduces the amount of the anisotropic energy and, eventually, thermal energy (~k_B_T) [8] might take over and randomize the orientations, which would imply the erasing of the information previously written. Therefore, reducing the bit size requires increasing the anisotropy of the material, and hence materials with higher K_u_ were investigated. At this point, the geometry of the bits changed from in-plane to out-of-plane [9] (see Figure 2A,B) as it facilitates reaching those higher fields and increasing the maximum areal density possible from around 40 Gbits/in^2^ to 100 Gbits/in^2^ [10]. A drawback of this perpendicular geometry is that it carries a higher magnetostatic energy [8] and therefore it is not naturally obtained unless a special material with perpendicular magnetic anisotropy (PMA) is employed [9,11]. In the quest for these PMA materials, RE/TM alloys and heterostructures offer many possibilities [12,13,14].

Therefore, the focus moved from traditional materials such as FePd or CoCrPt to materials with high magneto-crystalline anisotropy as CoPt or SmCo_5_ [15]. Sometimes these alloys can benefit from the addition of other elements, such as Cr and B on CoPt, that segregate and favor the creation of smaller bits and therefore higher recording densities [16]. RE–TM alloys are of interest because, as we will see below, they might help to increase the magnetic anisotropy and facilitate PMA. The higher coercivity of these materials also makes it more difficult to flip the bit, requiring a higher external applied field. To overcome this difficulty, a small diode laser next to the main pole locally heats the material near or above its Curie temperature to reduce coercivity, thus facilitating the flipping in the “heat-assisted magnetic recording” (Figure 2C). To facilitate a local heating as high as 10 K/nm, Au plasmonic antennas are of great help even though the high temperatures reached are a source of technical difficulties as well [17]. This is a promising technique to increase the areal storage density that has already proved feasibility [18]. In 2013, Wu et al. published the results of a non-commercial prototype of a hard disk using this technology reaching densities above 1 Tb/in^2^ [19].

In this work, we focus on the use of thin films and heterostructures of RE–TM alloys for applications. Hence, we will start in the following section with a general introduction to ferrimagnetism (Section 2) since it is the basis for all the other sections. In the subsequent sections, we will review some of the main applications of ferrimagnets such as spin valves (Section 3), the spin orbit torque (SOT) that opens the door to many spintronic applications (Section 4), moving domain walls (Section 5), and we will finish with a reflection on what the new standard for magnetic storage could be in the future: ultrafast all-optical switching (Section 6).

## 2. Ferrimagnetism

This particular kind of magnetic order has been behind many applications in the field of magnetic recording since the beginning, and it has recently attracted the attention of scientists because of its potential for applications in the near future, especially in thin film form.

**Figure 3:**
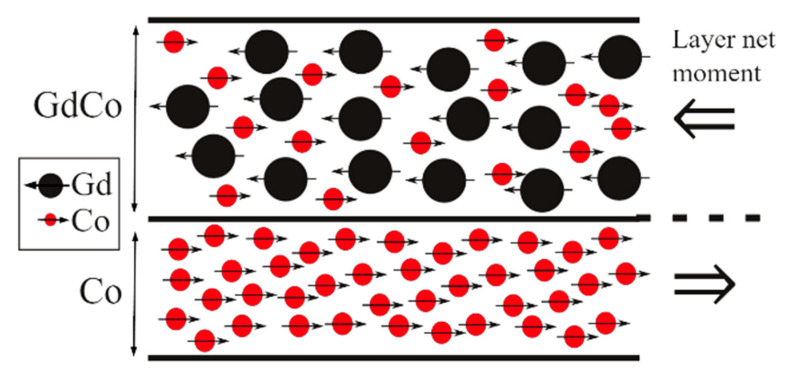
Schematic of a bilayer structure that is repeated 20 times to form a ferrimagnetic multilayer Si/[Gd_0.5_Co_0.5_/Co]_x20_. There is a double ferrimagnetic order: one of the layers is a Gd_1__−x_Co_x_ alloy and the whole multilayer shows a ferrimagnetic order as neighbouring layers point in opposite directions.

In ferrimagnets, we have two magnetic sublattices: sometimes because the material is made up of two types of magnetic atoms, sometimes because the same atoms have two different locations in the crystalline lattice that make them behave differently. Both sublattices couple antiferromagnetically, but as their moments are not equal, they cancel each other out only partially and there remains a non-zero macroscopic magnetization [20]. The order temperature below which this structure exists is known as the Curie temperature. Above it, they behave as paramagnets. In this short review, we will focus our attention on RE/TM structures in thin film, which is the preferred geometry for many applications in the field of information recording and sensors. For example, in Figure 3 the top layer is a Gd_1−x_Co_x_ ferrimagnetic alloy with two magnetic sublattices.

The magnetic electrons in lanthanide RE reside in the internal *4f* shell and are rather shielded by *5d* and *6s* shells. The non-spherical distribution of electrons in a *4f* shell gives these materials a large anisotropy. The exchange interaction between RE and TM in an alloy is not direct as it is in ferromagnets but through the coupling of the intermediate *5d* electrons of RE [21]. From the application of Hund’s rules, two groups can be distinguished: for light RE elements (from Ce to Eu) the *4f* shell is less than half filled and the coupling between RE and TM is parallel (ferromagnetic), whereas for heavy RE (Gd and above) the coupling is antiparallel, and ferrimagnetism shows up. We will concentrate here on this second case. The strong spin–orbit coupling of RE gives rise to a large local anisotropy, which is desirable for many applications as we will see. Gd is an exception because it has the *4f* shell only half-filled (7 electrons), and therefore it is an S ion (L = 0) and shows a small anisotropy [22]. Seminal examples of these structures were (Gd,Tb)/(Fe,Co) [23].

**Figure 4:**
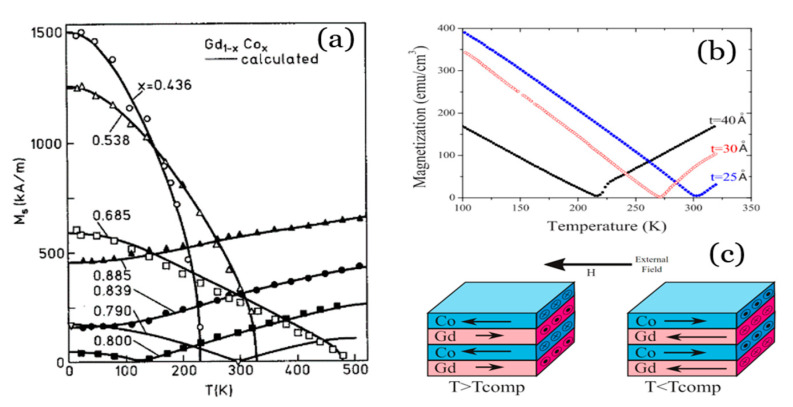
(**a**) Magnetization of GdCo alloys of several compositions. Reprinted with permission from ref. [23]. 1989 AIP. (**b**) Magnetization of GdCo/Co ferrimagnetic multilayers showing different T_comp_ depending on the thickness of Co layers (labeled in the graph). Reprinted with permission from ref. [25]. 2008 APS. (**c**) Outline of the aligned states of a Gd/Co multilayer at temperatures below and above T_comp_, where the dominating sublattice changes.

An important parameter in ferrimagnetic systems is the so-called compensation temperature (T_comp_). Given the different dependence of magnetism on temperature in the two sublattices within the ferrimagnet, and the fact that both point to opposite directions, sometimes there is a temperature at which both sublattices reach the same value and cancel out. At this temperature, the system therefore behaves like an antiferromagnet. The existence and value of this temperature is at the origin of the great importance that these materials might have in applications, as we will see. In the case of the ferrimagnetic alloy Gd_1−x_Co_x_, for example, T_C_ for Gd and Co sublattices are very different (293 K and 1400 K); for concentrations around x = 0.78 the compensation is present at room temperature. Figure 4a shows the dependence of the magnetization (M) of several Gd_1-x_Co_x_ alloys (0.44 < x < 0.8) with temperature and the different T_comp_ associated [23]. In each case, below T_comp_ the Gd sublattice is stronger and aligns with the field, forcing the Co sublattice to point antiparallel to the external field. Above T_comp_, the behavior is reversed (Figure 4c). The small values of M near this temperature facilitate the perpendicular anisotropy, and small changes in temperature allow the magnetic ordering to change a lot, which is very useful for many applications. The coercive field depends inversely on M and diverges at T_comp_, which is also of interest in some applications.

In the case of heterostructures such as bilayers and multilayers made up of RE and TM, the antiparallel coupling at the interfaces determines the magnetic structure of the whole material as well, yielding an artificial ferrimagnet that shares most of the properties of the alloys (see Figure 3 and Figure 4c). The additional freedom of choosing the thicknesses (and sometimes composition and doping [24]) of the layers provides further tunability: the composition in the case of the alloy corresponds to the ratio of thicknesses of the RE and TM layers, facilitating the positioning of T_comp_ at the desired value [25]. For example, in Figure 4b the compensation of magnetization is also achieved in ferrimagnetic multilayers by changing the thickness of the Co layers while the other is kept fixed. A drawback of these structures resides in the affinity of TM and RE for mix up at the interfaces [26], which, to make things worse, is asymmetric [27]. This smears out the properties, so much effort was directed towards the quantification and limitation of its extent. A successful strategy was replacing the RE layer by an alloy where RE still dominates the magnetization of that layer [28,29,30].

## 3. Spin Valves

A spin valve system typically consists of two ferromagnetic (FM) or ferrimagnetic (FiM) layers separated by a nonmagnetic layer, where the electrical resistance of the system changes between two values depending on the relative alignment of the magnetic layers: minimum when the layers are parallel and maximum when they are antiparallel. The interlayer exchange coupling is revealed in the sign and strength of the coupling between both FM (FiM) layers whereas the change in the resistance is due mainly to the GMR effect, although there can also be other MR contributions (as, e.g., AMR) [1,31]. This kind of system has been broadly used as magnetic sensors and in magnetic recording (as part of the reading head), as we pointed out before.

**Figure 5:**
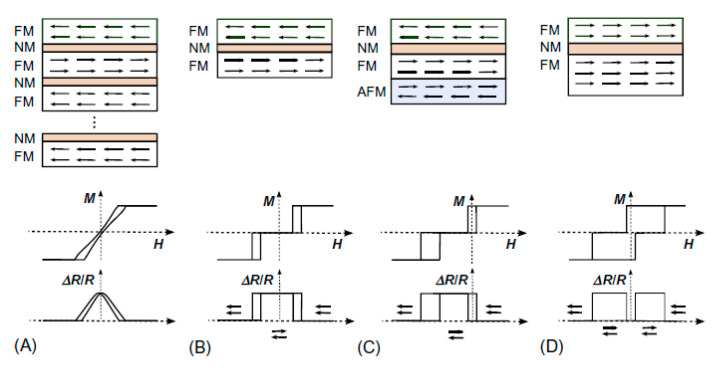
Schemes of several spin-valve configurations: Multilayer spin-valve, consisting of the repetition several times of two identical magnetic layers coupled antiferromagnetically (**A**); Bilayer spin valve, which consists of two identical magnetic layers coupled antiferromagnetically (**B**); Exchange bias spin valve, in which one of the magnetic layers is exchanged biased to an antiferromagnetic layer. Hence, the free layer on top can rotate nearly independently of the bottom layer, which acts as a reference/pinned magnetic layer (**C**); Pseudo spin-valve, consisting of a magnetically soft layer that can rotate nearly freely in relation to a hard ferromagnetic layer (which can be a different and/or a thicker ferromagnetic layer) (**D**). In each panel, the corresponding sketches of the hysteresis loops (middle) and the relative change in magnetoresistance (bottom) are also shown. Reprinted with permission from ref. [1] with permission from 2018 Elsevier.

In Figure 5, we show several types of GMR spin valves [1,31] developed after the discovery of the GMR effect. The *bilayer spin valve* (Figure 5B) is essentially like the typical spin valve described previously. If we tune its magnetic properties, it is possible to optimize the magnetic response, allowing us to obtain two possible bit states, “0” for antiferromagnetic (AF) orientation (maximum resistance) and “1” for ferromagnetic (FM) orientation (minimum resistance), well-suited for magnetic recording and memory. In the case of the *multilayer spin valve* (Figure 5A), the GMR is higher than in the bilayer one, as there are more interfaces contributing to the spin-dependent electron scattering phenomenon [1]. The fact that both magnetic layers are strongly AF coupled gives rise to noncollinear relative orientations in each layer, limiting its efficiency. Hence, the *exchange bias spin valves* (Figure 5C) were developed, in which an AF layer was grown close to one of the FM layers. The exchange interaction between these two layers prevents the moment of this FM layer (“pinned layer”) from following the external field (exchange bias mechanism), presenting therefore an unidirectional anisotropy and higher coercivity [32,33], whereas the other FM layer is practically free to follow the external applied field (therefore called “free layer”). Finally, another possibility is the *pseudo spin valve* (Figure 5D) [34], where the magnetic layers have different coercivities (i.e., one is a soft magnetic material and the other is a hard one), providing a window of field values in which the magnetization of the layers are opposites [1,35]. In that case, the soft magnetic layer is called the free layer and the hard one is called the fixed or pinned layer.

### Spin Valves with RE–TM Alloys

**Figure 6:**
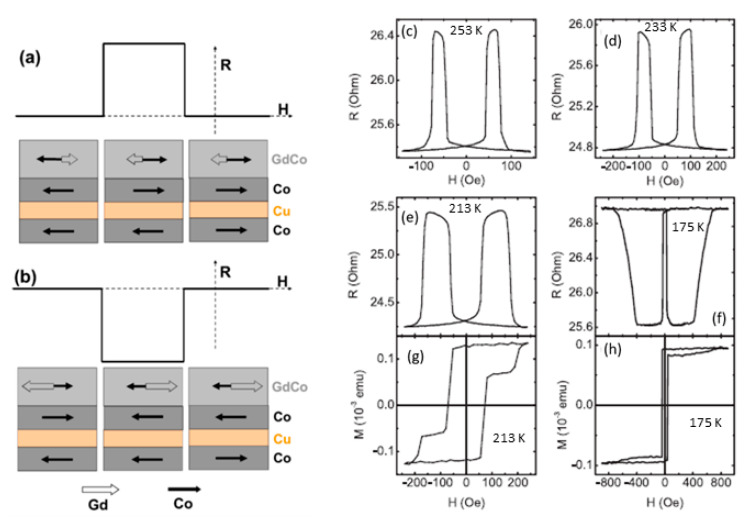
Schematic configurations of the magnetic moments of pure Co layers and Co and Gd sublattices of the Gd-Co layer, where the moment of Co total subnetwork is larger than that of Gd sublattices of the Gd-Co(35 nm)/Co(7 nm) composite layer at a temperature above the T_comp_ (**a**) and vice versa, at a temperature below T_comp_ (**b**). Magnetoresistance (**c**–**f**) and magnetic moment (**g**,**h**) loops for the Gd-Co(35 nm)/Co(7 nm)/Cu(4 nm)/Co(7 nm) spin valve measured at different temperatures: 253 K (**c**), 233 K (**d**), 213 K (**e**,**g**), and 175 K (**f**,**h**). The upper GdCo/Co bilayer act as the fixed layer in the spin valve, and the lower Co layer acts as the free one. Reprinted with permission from ref. [44] 2016 Elsevier with permission.

Many different materials have been used in spin valves, including of course RE–TM ferrimagnetic alloys and multilayers (e.g., [36,37,38,39,40,41,42,43]). For example, Bellouard et al. [36] obtained a spin valve formed by a CoFe/Ag/CoFeGd trilayer back in 1997 and observed an increase in the spin-valve effect when a thin CoFe layer was inserted between the Ag and CoFeGd layers. Most spin valves do not behave very differently when the temperature changes: they just rely on an external field with resistance. However, using RE/TM alloys and multilayers give us more freedom to play with, especially near T_comp_ where the dominant sublattice changes. For example, Jiang [39] studied spin valves with GdCo as the free layer, with a focus on the temperature dependence of current-induced magnetization switching of the free layer, finding a reversed current-induced alignment of the moments at temperatures close to the compensation (T_comp_). Other authors also observed temperature dependencies linked to T_comp_ of the FiM layers (e.g., the work by Bai et al. [41] with FeCoGd). Following this trend, Svalov et al. [44] proposed a thermo-sensitive spin valve based on a Gd-Co/Co/Cu/Co structure. In this case, the Gd-Co/Co bilayer is indeed an artificial ferrimagnet acting as the fixed layer of the spin valve, and the upper Co layer as the free one. In Figure 6 the schematic configurations of the magnetic moments and the magnetic and magnetoresistance loops are shown. In this case, the T_comp_ of the layered artificial ferrimagnet is 180 K. Above T_comp_, the magnetic moment of the Co sublattice dominates over that of the Gd sublattice and determines the direction of the total magnetic moment of the composite layer. Therefore, a large magnetic field aligns the Co moment of the bilayer with that of the free layer (see Figure 6a). Hence, at 253 K and H = −45 Oe, a small magnetic field, the moment of the free layer becomes antiparallel to the fixed layer and the resistance increases (see Figure 6a,c). At H = −75 Oe, the magnetization reversal of the fixed layer occurs, decreasing the resistance again. Below T_comp_, the Gd sublattice dominates over the Co one. Hence, when a large magnetic field is applied, the Gd moment aligns parallel to that of the Co free layer, whereas the Co moment of the Gd-Co/Co bilayer is aligned antiparallel to the Co free layer moment, giving rise to a negative MR (see Figure 6b,f). Hence, this kind of spin valve could be used as a switch triggered at a certain temperature [44], which might be useful, for example, in position sensors [45,46]. In the same vein, Milyaev et al. [47] also found a certain temperature-induced switching between low- and high-resistance states, in a Ta/Gd/Co_90_Fe_10_/Cu/ Co_90_Fe_10_/Fe_50_Mn_50_/Ta spin valve (i.e., a conventional exchange-biased spin valve with the insertion of a Gd layer). The trigger temperature of the switch depends on the T_comp_ of the CoFe/Gd artificial FiM and, therefore, can be controlled by the thickness of the Gd layer. Hence, this kind of valve could be used for magnetic recording (with a current providing both the magnetic field and the heating required for the switch).

**Figure 7:**
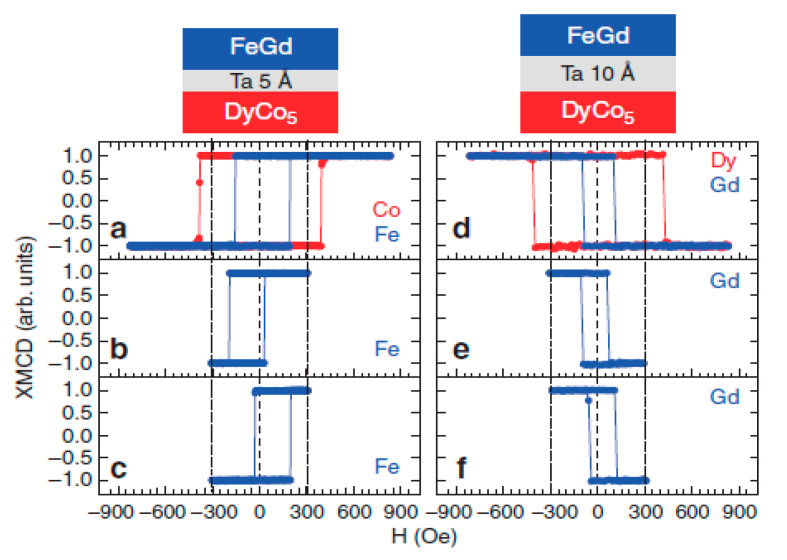
Exchange-biased loops for two representative samples. Left column: hysteresis loops measured for a sample with 0.5 nm Ta spacer: measured from 900 to −900 Oe (**a**), measured between 300 and −300 Oe after saturating at 3 kOe (**b**), idem to (**b**) but after saturating at −3 kOe (**c**). Right column: the same scenario was followed for a sample with a 1 nm Ta spacer (**d**–**f**). The dotted vertical lines mark the 300 and −300 Oe fields. Reprinted with permission from ref. [52] 2012 Nature.

Another related aspect that could be worthy of study in the close future is the appearance of giant exchange bias shifts (of up to tens of kOe) in ferrimagnetic/ferromagnetic [48,49,50] and ferrimagnetic/ferrimagnetic [51] bilayers, which could be extremely useful for spin valves and other spintronic applications. Unfortunately, these exchange bias phenomena are usually observed at low temperatures, which decreases its potential for applications (usually requiring a working range of temperatures close to room temperature). In that sense, the ferrimagnetic spin valve proposed by Radu et al. [1,52], presenting exchange bias phenomena at room temperature and without needing a field-cooling protocol, seems like something that deserves more study, given its potential for applications. In particular, Radu et al. [52] fabricated a FeGd/Ta/DyCo_5_ spin valve, where DyCo_5_, (with high T_C_ and strong anisotropy) is the hard layer and a ferrimagnetic FeGd alloy (with low T_comp_) is the soft one. Both have rectangular hysteresis loops, and in this case, the interlayer is used to only partially decouple the FiM layers (contrary to the standard case, where the decoupling is total) and this partial decoupling allows the existence of perpendicular exchange bias [52]. In Figure 7, exchange-biased loops for two samples are shown. Noteworthily, these exchange bias phenomena are observed at room temperature and without requiring a field-cooling procedure. Even more, there is no training effect (i.e., the exchange bias fields remain stable after measuring several hysteresis loops). Therefore, this FiM spin valve could be really useful for applications in ultrafast storage media [53].

With regard to the application of spin valves to magnetoresistive random access memory (MRAM), Iusipova [54] recently conducted a study of the switching characteristics of spin valves with longitudinal anisotropy and found that one of the most promising materials for such an application was Co_80_Gd_20_ alloy (annealed at 200 °C) because of its high spin polarization parameter, which lowers the magnetic switching field, showing the potential of this kind of alloy.

The dynamic properties of spin valves have rarely been studied, in spite of their relevance for high-frequency spintronic applications [55]. In particular, the use of spin valves in magnonic devices is of limited use given the lack of studies of their dynamics. Hence, Chen et al. [56] have studied the interlayer transmission of magnons in spin valve structures using the magneto-optical Kerr effect and have found that the insertion of RE layers increases the interfacial dissipation of magnons. On the other hand, Zhang et al. [55] have observed a selective tuning of the Gilbert damping constant in a NiFe/Cu/CoFe spin valve by inserting different RE (Gd, Tb) nanolayers to the FM layers due to the enhanced orbital moment of Ni and Co, the spin and orbital moments of the RE, the electronic band structure of the TMs, and the lattice structure. This opens the possibility of designing optimized spin valves for high-frequency spintronic devices.

## 4. Spin–Orbit Torque Devices

Until the 90’s, electronic circuits were based their operation on the *charge* of the carriers, usually electrons. Taking advantage of the fact that electrons have an intrinsic moment (*spin*) in addition to its charge, *spintronics* [17] has recently emerged as an improved electronic technology that makes use of both the charge and the spin of electrons. This provides more functionalities to devices, lower power consumption in general, the possibility of building non-volatile devices, and greater scalability [17].

An important advance that spintronics provides is the ability to manipulate the magnetic moment of a ferromagnetic material in a purely electrical way. Some materials and structures can separate (filter) electrons as a function of their spin orientation, building currents of spin-polarized electrons [57]. When these electrons pass through a magnetic material, they act on its magnetic moments and can eventually switch its moment without an externally applied magnetic field. Apart from the multiple functionalities that this offers, it also reduces the power consumption. The first spintronic effect of this kind, studied in 1996, was spin-transfer torque (STT) [58,59]. In the STT effect, a change in the magnetic orientation of a ferromagnet is induced by a spin-polarized current. STT can be used to build magnetic memories such as magnetoresistive RAM using a magnetic tunnel junction. The basic structure of STT devices consists of an oxide tunnel barrier sandwiched between two FM layers. When the current passes through one of them, it becomes spin polarized and in turn this changes the magnetic state of the other FM layer. However, these STT devices require high currents to manipulate the magnetization and can lead to a breakdown of the oxide layer [60]. Therefore, substantial efforts are still being devoted to reducing the amount of current required for manipulating the magnetization using this effect. In particular, and over the last decade, an alternative to STT has been developed, the spin–orbit torque (SOT) [61], which does not require such high currents.

**Figure 8:**
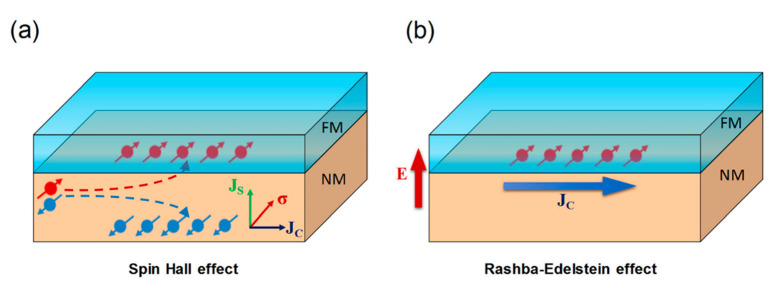
Schematic illustration of the bulk spin Hall effect in a heavy metal (**a**) and the interfacial Rashb–-Edelstein effect (**b**). Reprinted with permission from ref. [64] 2018 APS.

The simplest SOT device consists of a bilayer made of a non-magnetic metal (NM) and a ferromagnet (FM). When an electrical current flows in the plane of the NM thin film, a transverse spin current is generated due to the spin–orbit coupling. This causes an accumulation of spin in the NM/FM interface that exerts a torque on the magnetization of the FM. The detailed mechanism that causes this accumulation of spins at the interface is still under debate, but basically it combines the spin Hall effect (SHE) and the interface Rashba–Edelstein effect [62,63,64]. In the spin Hall effect, an unpolarized current passes through a material with a high bulk spin–orbit coupling (SOC). Electrons with different spin are deflected in opposite directions; this deflection between spin-up and spin-down creates a transverse spin current and generates a spin accumulation at the NM/FM interface (Figure 8a). This bulk effect in the NM arises from the band structure of the metal and can also be caused by the presence of high SOC impurities. On the other hand, the Rashba–Edelstein is an *interfacial* SOC phenomenon. In structures with broken symmetry (e.g., bilayers of different materials), an electrical field is generated perpendicularly to the interface. Electrons moving close to this interface experience an effective magnetic field that couples to the spin of the conduction electrons and polarizes their moments parallel to the interface (Figure 8b).

The basic structure of a SOT device consists of a conductive layer of non-magnetic material (NM), the magnetic layer to be handled, and a final protective layer. Compared to other spintronics devices, SOTs offer the advantages of lower structural complexity, low cost, ultrafast response, and power efficiency. Heavy metals (HM) such as Ta, W, Pt, or Hf are normally used as NM materials due to their high spin–orbit coupling.

For the magnetic layer, in turn, the use of materials with perpendicular magnetic anisotropy (PMA) is preferred, so they make SOT-induced switching more efficient. The most commonly used is ultrathin CoFeB capped with MgO. The role of this capping layer of MgO, employed with other ferromagnets as well (Co, Permalloy -NiFe-, or CoFe), is not only to protect against oxidation but to originate the PMA thanks to the hybridization at the FM/MgO interface [65]. The interfacial nature of this anisotropy severely limits the thickness of the layers (to the order of 1 nm) and requires having a correct oxygen stoichiometry as well as a very clear interface. Such small thicknesses impose limitations on the scaling of SOT devices. For applications such as MRAM, the thermal stability is proportional to the magnetic volume and thin layers are not desirable. Magnetic multilayers have also been employed as Co/Pt [66] or Co/Pd [67]. They can keep their PMA up to higher thicknesses but at the cost of increasing the complexity of the SOT device.

### 4.1. RE–TM Ferrimagnetic Alloys in SOT Devices

In the first SOT devices studied, RE were not employed: just TM for both layers. In recent years, though, there has been a growing interest in replacing FM with materials with negative exchange coupling, such as ferrimagnets and antiferromagnets. In particular, RE–TM ferrimagnetic alloys have been proven to be ideal candidates because of their versatility to vary their magnetization with composition, and because they exhibit PMA even in bulk form due to different reasons [68,69,70,71]. This is the case especially near T_comp_, because of the reduced magnetostatic energy associated with a small magnetization due to the partial (or total) cancellation of the moments of the two sublattices that form the material. This in turn allows for the use of larger thicknesses while keeping PMA.

In 2017, Mishra et al. [72] showed for the first time that using a GdCo alloy as the magnetic layer in a SOT device greatly increased the efficiency of the effect near the magnetic compensation point. Although SOT scales inversely with saturation magnetization, they surprisingly observed an anomalous increase in the SOT efficiency close to the compensation temperature in this case. They noticed that for alloys with concentrations close to magnetic compensation at room temperature, when the magnetization decreases by a factor of two, the longitudinal SOT effective field and switching efficiency are increased six and nine times, respectively. The reason for this behavior is that the negative exchange interaction provides an additional torque that in turn increases the SOT effect. This additional torque is caused by the negative exchange interaction between the ferrimagnetic sublattices in the alloy. This improvement in SOT efficiency is more accentuated in the GdCo alloy because of the collinear character of the antiparallel coupling between the Gd and Co sublattices.

**Figure 9:**
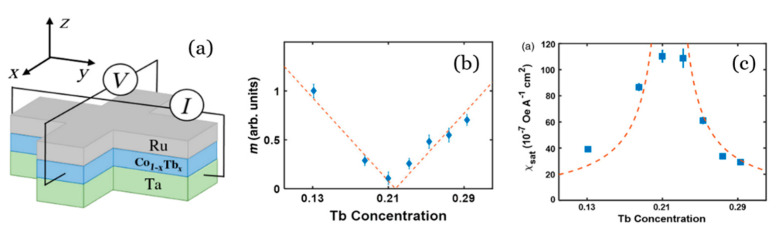
(**a**) Schematics of the device geometry for the measurement of anomalous Hall resistance. (**b**) Magnetic moments of Co_1−x_Tb_x_ alloys as a function of Tb concentration showing magnetic compensation. (**c**) Saturation SOT efficiency for different Tb concentration. Reprinted with permission from ref. [75] 2016 APS.

Using GdCoFe alloys with concentrations that present magnetic compensation at room temperature and PMA, Roschewesky et al. [73,74] have achieved interfacial torque in ultra-large thickness layers (up to 30 nm) with high thermal stability. The ability to switch magnetization in such thick ferrimagnets while keeping a high thermal stability might have important implications for future applications of SOT devices. Other ferrimagnetic alloys, based on Tb instead of Gd, also show an increase in SOT efficiency close to T_comp_ (see Figure 9) [75]. It has been shown that in these ferrimagnetic RE–TM alloys, the SOT switching in combination with the fast magnetic dynamics and minimal net magnetization promises faster spintronic devices than those with traditional ferromagnetic systems. Figure 9a shows the typical way to characterize the SOT effect using the extraordinary (or anomalous) Hall effect. The electric current goes through the heavy metal (Ta) and the voltage is measured in the film plane but perpendicularly to the polarized current.

### 4.2. SOT Using Topological Insulators

**Figure 10:**
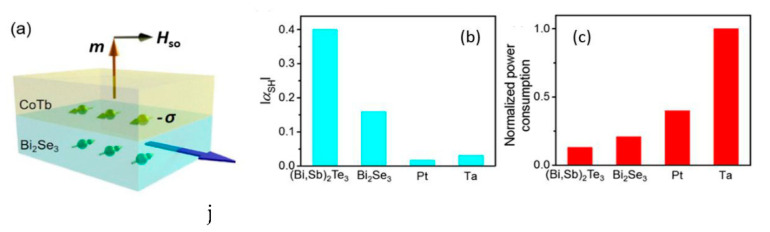
(**a**) Schematic heterostructure TI/RE–TM alloy. The charge current *j_e_* generates spin accumulation (−σ) that is perpendicular to the current direction at the interface and exerts a spin–orbit torque on the magnetic moment in the CoTb PMA alloy. (**b**,**c**) Absolute values of the effective spin Hall angle and power consumption of different spin–orbit materials, showing the higher efficiency of TI vs. traditional heavy metals such as Ta and Pt. Reprinted with permission from ref. [76]. 2017 APS.

In addition to metals, the NM layer has been proposed to be made up of topological insulators (TI). They are a new class of materials with a strong spin–orbit coupling and are therefore good candidates to replace heavy metals in SOT heterostructures because the current density needed for the switching is more than one order of magnitude smaller. Han et al. reported spin–orbit torque switching in heterostructures employing Bi_2_Se_3_ as TI and a CoTb ferrimagnetic alloy on top of it. CoTb gets the optimal PMA thanks to the TI and, for the first time, they observe efficient SOT switching induced by a TI at room temperature [76] (see Figure 10). More recently, Wu et al. have been able to tune the Gd concentration in GdCoFe alloy to significantly enhance the SOT efficiency (up to 6.5 times) working near T_comp_, and reaching switching speeds of a few picoseconds [77]. Therefore, SOT heterostructures based on topological insulators and RE–TM ferrimagnetic alloys offer a promising route for practical, energy efficient, and high-speed spintronic devices.

### 4.3. Examples of SOT-Based Sensors

**Figure 11:**
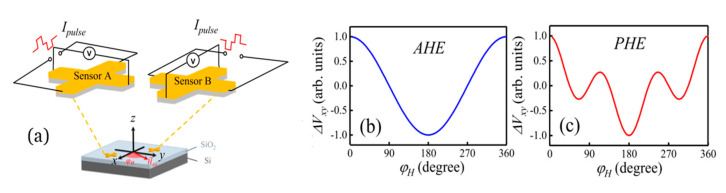
(**a**) Angular sensor composed of two identical Hall crosses orthogonally placed on the same substrate to allow 2D measurement of the magnetic field. In order to get an accurate result, the extraordinary (**b**) and planar (**c**) Hall contributions have to be separated. Reprinted with permission from ref. [79] 2018 AOP.

We can find a practical application of a SOT device in angular position sensors. This type of sensors has a wide range of applications in manufacturing, consumer products, and automotive engineering [78]. As stated above, many kinds of MR sensors have been developed in the last 50 years to probe magnetic fields, as in the reading heads of hard disks. Depending on the orientation of the sensor and the external field, they yield a sinusoidal output signal. Thus, these devices can act as an angular position sensor as well. However, they usually require complicated structures (magnetic multilayers) and complex manufacturing processes that make them more expensive than traditional Hall sensors. Hence, Luo et al. [79] have recently designed an angular position sensor based on SOT (see Figure 11). It consists of two orthogonally positioned Hall crosses to measure the X and Y axis simultaneously and compose the 2D vector. The structure they employ is a heavy metal/ferromagnet (HM/FM) bilayer (in particular, Pt_3nm_/Co_3nm_), and they obtain an average angle error of only 0.65° over 360° from the prototype device. This design is already simpler and cheaper than its MR counterpart, but its design could be further improved by optimizing the choice of materials, for example using RE–TM alloys instead of Co, as we might tune the magnetic parameters (saturation magnetization, coercivity, temperature dependence, etc.) with a proper election of RE and composition.

Since the magnetization switching driven by spin torque depends on the longitudinal magnetic field, Xie et al. [80] have proposed very recently a magnetic field sensor based on a SOT device. It has major advantages over traditional MR sensors, which require a magnetic bias to achieve a linear response to the external field, while the SOT sensor can achieve the same without any magnetic bias. This greatly simplifies the sensor structure. In addition to this, when operating with ac current in the new design, the dc-offset is automatically suppressed, and no compensation circuit is needed. They experimentally demonstrated that their SOT sensor can work linearly in the range of 3–10 Oe with negligible hysteresis and dc offset. This good linearity is almost 10 times larger than that of a typical MR sensor. Moreover, the output voltage is about one order of magnitude larger than that of MR. These advantages, combined with its extremely simple structure, make the SOT-based sensor well suited for replacing the more expensive MR sensor in the near future.

## 5. Domain Wall-Based Devices

Another technological forefront in which RE/TM ferrimagnets are playing an important role is the movement and manipulation of domain walls (DW) by using electrical fields instead of magnetic ones (the original way to do it). Back in the 1980’s, Berger et al. proposed and experimentally demonstrated the possibility of manipulating magnetic DW using electrical currents instead of magnetic fields [81,82]. When a polarized electrical current crosses a DW, the coupling between the spin-polarized electrons and the local magnetic moments in the DW induces a torque on the DW. For applications, the two interesting parameters to deal with are the threshold current at which DW movement starts and their velocity. Compared to FM systems, structures with antiparallel coupling show faster spin dynamics. Therefore, RE–TM ferrimagnetic alloys are potential candidates for high-speed devices based on DW motion [83].

**Figure 12:**
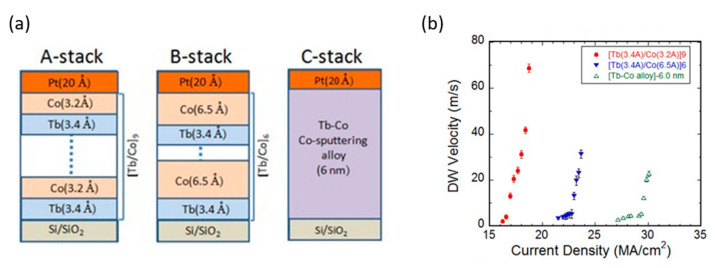
(**a**) Different Tb–Co ML and alloy structures with perpendicular magnetic anisotropy, (**b**) Current density dependence of DW velocity for magnetic wires with these structures. Reprinted with permission from ref. [84] 2016 APS.

The critical current strongly depends both on the composition and on the layered structure. Bang et al. [84] have investigated the current-induced domain walls in wires of Tb/Co multilayers and alloys with PMA. Figure 12 shows the strong dependence of both the critical current and the DW velocity on different configurations of the same materials they studied. Better properties are observed for thin Co layers and large number of repeats (interlayers), as a lower critical current and higher DW velocities are obtained in this case (A-stack) compared with the case of the pure alloy (C-stack). They suggest that the enhancement of the efficiency they find arises from the skew scattering associated with the Tb impurities present in the Co layers. This study presents an efficient way to reduce the critical current density for DW motion through simple structural engineering using RE–TM alloys.

Recently, ultrafast field-induced DW motion has been observed in GdFeCo alloys close to their magnetic compensation [85,86]. Since in the RE–TM ferrimagnetic alloys RE and TM moments can be easily controlled by temperature or varying the composition, these alloys are good candidates for DW motion-based applications. Compared to FM systems, RE–TM alloys present a threshold current density one order of magnitude lower, higher DW velocities, and lower power consumption. An important effect should be especially taken into account in these systems: Joule heating. The currents employed to control magnetization generate heat that increases the temperature of the material, changing in turn the values of some working parameters. As being close to the compensation temperature is also of great importance in these materials, the currents have to be kept low enough to keep this temperature change within a few degrees only [87].

DW manipulation with electric currents has aroused great interest for its possible applications as well. Parkin proposed the use of the controlled movement of DW in magnetic nanowires by short pulses of spin-polarized currents for innovative memory devices, known as racetrack memories [88]. In this technology, the information is stored in a series of domain walls arranged in a 3D array of nanowires that are moved along with polarized currents. The system has no moving parts and is essentially 3D [89], unlike traditional data storage, which is 2D. It has been evolving since it was proposed 15 years ago, and it is getting closer to its practical realization. The latest proposals are based on synthetic antiferromagnets reaching velocities of movement of the domain walls around 1 km/s [90].

Concerning most of these applications where the maximization of an effect is achieved at the compensation temperature, it must be pointed out that there are in fact two compensation temperatures usually defined: the magnetic compensation temperature (T_M_) where the magnetic moments (m_i_) are balanced; and the angular momentum compensation (T_A_), where the angular momenta (A_i_) of both species cancel out. As $m_{i}=A_{i}\cdot g_{i}\cdot {µ}_{B}/\hbar$, both are not the same due to the different values of the Landé factors. For example, for Gd-Co, g_Co_ = 2.2 and g_Gd_ = 2.0, which yields a difference between them of ~10 K [91]; while in Tb-Co the difference reaches ~50 K [92]. Different applications maximize at T_A_ [93,94] or T_M_ [91,92] depending on the microscopical origin of the key interaction involved. There is still a lot of research to be done on this topic. In order to determine T_A_, dynamical techniques such as X-ray magnetic circular dichroism (XMCD) are needed, although other more accessible techniques based on the analysis of critical exponents’ behavior have been proposed [95].

Other future applications of these DW structures are electronic devices such as spintronic memristors [96] or DW logic gates and circuits [97]. Nowadays, thanks to the possibility of manufacturing nanosized structures, more complex applications are being found, such as an ingenious turn counter devices based on DW motion in a square spiral nanotrack. This sensor has been developed by Mattheis et al. and has been commercialized by Novotechnik [98,99].

## 6. All Optical Switching

In addition to the interest that TM/RE alloys and heterostructures have definitively had in both fields of fundamental study of magnetic interactions and magnetic recording applications, these materials have recently drawn a renewed interest as promising candidates for future technologies. Indeed, the quest for higher densities of information storage, faster access, and less power consumption requires a new paradigm to be developed in the field of data storage. Again, TM/RE structures are here called to play a significant role.

Writing information in a bit requires applying an external magnetic field to make sure that the moment of that bit finishes pointing in the direction of that field. This can be done essentially in two ways: with an external field in the same direction of the moment, which is the easy axis, making the domains move; or with a field applied at 90° of this direction, which causes a precession of the moment around the hard axis. The dynamics of the latter method, known as precessional switching, turns out to be the fastest [100] and therefore is preferred [101,102]. The angle rotated by the moment around the external field is proportional to the product of the external field and the duration of the pulse. The switching usually takes ~10–100 ps [62]. In order to make the switching faster, this time has to be reduced and this might be achieved by increasing the strength of the field, although there is an upper limit to the field that can be applied [103].

**Figure 13:**
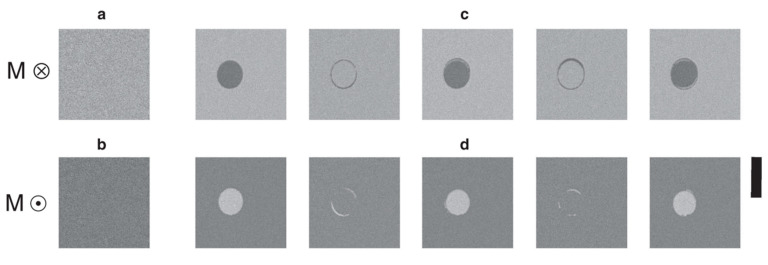
The magneto-optical images of a Gd_24_Fe_66.5_Co_9.5_ continuous film obtained after the action of a sequence of N 100 fs laser pulses. (**a**,**b**) Initial homogeneously magnetized state of the film with magnetizations ‘up’ and ‘down’ as represented by the circled dot and cross, respectively. The light grey region represents magnetization pointing ‘down’ and the darker grey ‘up’. (**c**,**d**) The film after excitation with N (N = 1, 2, …, 5) pulses with a fluence of 2.30 mJ·cm^−2^. Each laser pulse excites the same circular region of the film and reverses the magnetization within it. The scale bar on the right corresponds to 20 µm. Reprinted with permission from ref. [105] 2012 Springer Nature.

The development of ultrafast lasers opened the possibility of exploring the dynamics of magnetism at the time scales of pico and femtoseconds. The first experimental observation of such a phenomenon is due to Beaurepaire in 1996 [104], who employed sub-picosecond pulses of laser on a Ni ferromagnetic layer to demagnetize it. In 2007, Stanciu [100] demonstrated that switching deterministically a layer of ferrimagnetic GdFeCo alloy was possible without any magnetic applied field just by means of a single 40 fs laser pulse. This is known as “all-optical switching” and it is at least 1000 times faster than using precessional switching, so it would have interesting potential if it could be technologically applied to data storage. A bit pattern could be written by scanning the surface of one of these materials with a femtosecond laser whose helicity is modulated by the binary sequence of “up” and “down” bits to write (see Figure 13).

This GdFeCo alloy was chosen because of its strong Faraday rotation that facilitates following the dynamics of the spins by using optical magnetometers, but also because it was thought that the polarization of the laser beam had the ability to act on the spins via the inverse Faraday effect, in a similar way to the external field, therefore inducing the switching. In 2012 and 2013, the same effect was found in different RE–TM alloys such as TbFe [106] or TbCo [107,108,109]. In 2014 it was extended to multilayers and other heterostructures such as Pt/Gd/Co trilayers [110] and others without RE, but always with ferrimagnetic coupling [111]. It is important to note that not only the duration of the laser pulse is important, but its fluence as well; and the switching is optimized when the temperature is just below the compensation temperature of the ferrimagnetic structure [112]. Optical switching is not exclusive to RE/TM systems. In 2017 it was demonstrated on thin Co/Pt and Co/Pd multilayers with PMA, and in Co/Ni multilayers [113] but they remain a minority so far.

The original explanation of all optical switching relied on a two-step process. First of all, the local ultrafast heating of the material makes it very susceptible to the field, and then the circularly polarized light pulse leads to the reversal via the inverse Faraday effect. This might indeed be the best explanation for a range of materials where helicity is required, but in 2012 Ostler [105] demonstrated that polarization of the beam is not necessary in the seminal GdCoFe system, as they showed that the switching could be achieved using a linearly polarized laser, just through the heat. In other systems such as TbCo alloys [108] or Pt/Co multilayers [114] the photon helicity is indeed required. Therefore, two kinds of all-optical switching are distinguished [115,116,117]: helicity-dependent and helicity-independent (also known as “all thermal switching”). Typically, the helicity dependent process requires several pulses, while for the thermally induced a single one is enough. The two-step explanation that Stanciu originally proposed is closer to that of the helicity dependent materials: a multi-domain formation due to the heating (for which helicity is not needed) followed by a re-magnetization of the material depending on the helicity of the laser pulse. This procedure usually requires a few pulses to ensure the switching [116], and even though the width of the pulse is of only a few femtoseconds, the total process of switching might take a few tens of milliseconds. Oddly enough, this explanation, which was valid for some of the cases, was not the one that applied in his case.

The counterintuitive physics behind the helicity independent switching has been a subject of study, as it was broadly thought that a scalar magnitude, such as temperature, could not determine the orientation of a vector magnitude, which the magnetic moment is. Although the fundamental mechanisms underlying the purely thermal switching are still under debate, it seems clear that the key lies in the different dynamics of the moments of the two sublattices of the material on ultrafast time scales [53]. The system has been studied from the point of view of the interplay between three energy reservoirs: electrons, lattice, and spins. The laser pulse produces a high temperature in the electron bath in the first 500 fs. This energy is transferred to the lattice bath through spin–phonon interactions on a longer time scale (1–10 ps) and then to the spin lattice. All these processes are strongly out of equilibrium and have to be studied separately to understand the full process [104,118].

**Figure 14:**
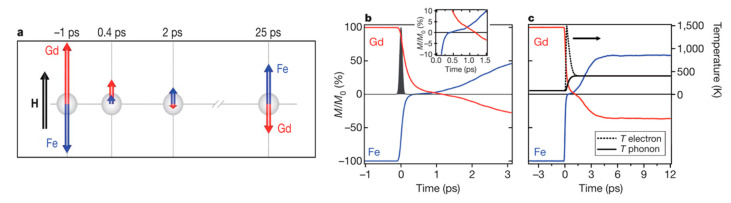
Computed time-resolved dynamics of the Fe and Gd magnetic moments from the localized atomistic spin model. (**a**) Cartoon-like illustration of the non-equilibrium dynamics of the Fe and Gd magnetizations with respect to an external magnetic field H. Simulated dynamics for the first 3 ps (**b**) and the first 12 ps (**c**) after laser excitation. As can be clearly seen, the demagnetization of the Fe is much faster than that of the Gd (see inset in b; axes same as main panel). For a time of 0.5 ps, we observe a parallel alignment of the magnetizations of the sublattices. The agreement with the experimental data is qualitatively excellent. Reprinted with permission from ref. [53]. 2011 Springer Nature.

In particular, Radu found that after the optical excitation and during the fast process of thermal switching, the ferrimagnetic structure passed through a ferromagnetic state due to a faster response of the TM sublattice regarding the RE sublattice (see Figure 14). TM moment sublattice collapses in 300 ps, while the RE sublattice takes about 1500 ps (~five times slower) to do so [53]. In fact, TM sublattice starts growing again while RE is still going down, and during a certain period of time both are aligned parallel although the exchange interaction between them is negative (antiparallel). He employed X-ray magnetic circular dichroism (XMCD) to have magnetic sensitivity and element specificity at the same time. In this way, the individual evolution of each sublattice was followed independently and that counterintuitive ferromagnetic-like state was found. It must be this intermediate ferromagnetic-like state that allows for the switching, as there are no other symmetry-breaking effects that can account for the deterministic toggle of magnetization. The times associated with the transfer of heat between the above-mentioned reservoirs (electrons-spins-lattice) and the width of the laser pulse are the key for the switching to happen [119]. The switching occurs for every single pulse (as opposed to helicity dependent processes that require a few). They also report that the laser fluence needs to be slightly above the window of values for the helicity dependent process of ref [112].

In order to technologically apply this phenomenon to data storage, many challenges are still ahead. One of them is the spot size of the laser, which is still too large to allow a competitive density of bits. A possibility to overcome this is the growth of Au nanoantennas next to the magnetic TbFeCo material to intensify the signal of the laser [120].

## 7. Conclusions

In summary, we have reviewed the interesting properties of RE/TM ferrimagnetic materials in thin film form that have played a significant role in the development of the data storage technology during the last 50 years. We have summarized new phenomena related to spintronics and ultrafast optical switching that could well be the future of this technology, and we find again these interesting materials in the forefront for new applications to store data faster, with less energy consumption and higher densities.

## References

[1] Radu F., Sánchez-Barriga J. (2018). Ferrimagnetic Heterostructures for Applications in Magnetic Recording.

[2] Grünberg P., Schreiber R., Pang Y., Brodsky M.B., Sowers H. (1986). Layered Magnetic Structures: Evidence for Antiferromagnetic Coupling of Fe Layers across Cr Interlayers. Phys. Rev. Lett..

[3] Baibich M.N., Broto J.M., Fert A., Van Dau F.N., Petroff F., Eitenne P., Creuzet G., Friederich A., Chazelas J. (1988). Giant magnetoresistance of (001)Fe/(001)Cr magnetic superlattices. Phys. Rev. Lett..

[4] Van Den Berg H.A.M., Clemens W., Gieres G., Rupp G., Schelter W., Vieth M. (1996). GMR sensor scheme with artificial antiferromagnetic subsystem. IEEE Trans. Magn..

[5] Amaral J., Pinto V., Costa T., Gaspar J., Ferreira R., Paz E., Cardoso S., Freitas P.P. (2013). Integration of TMR sensors in silicon microneedles for magnetic measurements of neurons. IEEE Trans. Magn..

[6] Iwasaki S.I. (2012). Perpendicular magnetic recording - Its development and realization. J. Magn. Magn. Mater..

[7] Weller D., Moser A. (1999). Thermal effect limits in ultrahigh-density magnetic recording. IEEE Trans. Magn..

[8] Chikazumi S., Graham C.D. (2009). Physics of Ferromagnetism.

[9] Piramanayagam S.N. (2007). Perpendicular recording media for hard disk drives. J. Appl. Phys..

[10] Choe G., Zhou J.N., Demczyk B., Yu M., Zheng M., Weng R., Chekanov A., Johnson K.E., Liu F., Stoev K. (2003). Highly in-plane oriented CoCrPtB longitudinal media for 130-Gb/in2 recording. IEEE Trans. Magn..

[11] Johnson M.T., Jungblut R., Kelly P.J., den Broeder F.J.A. (1995). Perpendicular magnetic anisotropy of multilayers: Recent insights. J. Magn. Magn. Mater..

[12] Ding M., Poon S.J. (2013). Tunable perpendicular magnetic anisotropy in GdFeCo amorphous films. J. Magn. Magn. Mater..

[13] Sajieddine M., Bauer P., Bruson A., Marchal G. (1996). Perpendicular magnetic anisotropy in an annealed Fe/Tb multilayer: A57Fe Mössbauer study. Solid State Commun..

[14] Tudu B., Tiwari A. (2017). Recent Developments in Perpendicular Magnetic Anisotropy Thin Films for Data Storage Applications. Vacuum.

[15] Sayama J., Mizutani K., Asahi T., Osaka T. (2004). Thin films of SmCo5 with very high perpendicular magnetic anisotropy. Appl. Phys. Lett..

[16] Lu B., Weller D., Sunder A., Ju G., Wu X., Brockie R., Nolan T., Brucker C., Ranjan R. (2003). High anisotropy CoCrPt(B) media for perpendicular magnetic recording. J. Appl. Phys..

[17] Hirohata A., Yamada K., Nakatani Y., Prejbeanu L., Diény B., Pirro P., Hillebrands B. (2020). Review on spintronics: Principles and device applications. J. Magn. Magn. Mater..

[18] Seigler M., Challener W., Gage E., Gokemeijer N., Ju G., Lu B., Pelhos K., Peng C., Rottmayer R., Yang X. (2008). Integrated near field transducer heat assisted magnetic recording head: Design and recording demonstration. IEEE Trans. Magn..

[19] Wu A.Q., Kubota Y., Klemmer T., Rausch T., Peng C., Peng Y., Karns D., Zhu X., Ding Y., Chang E.K.C. (2013). HAMR areal density demonstration of 1+ tbpsi on spinstand. IEEE Trans. Magn..

[20] Cullity B.D., Graham C.D. (2009). Introduction to Magnetic Materials.

[21] Campbell I.A. (1972). Indirect exchange for rare earths in metals. J. Phys. F Met. Phys..

[22] Hansen P. (1991). Chapter 4 Magnetic amorphous alloys. Handb. Magn. Mater..

[23] Hansen P., Clausen C., Much G., Rosenkranz M., Witter K. (1989). Magnetic and magneto-optical properties of rare-earth transition-metal alloys containing Gd, Tb, Fe, Co. J. Appl. Phys..

[24] González J.A., Andrés J.P., López de la Torre M.A., Riveiro J.M. (2001). Magnetic properties of thin film GdCoRe amorphous alloys. J. Alloys Compd..

[25] Andrés J.P., González J.A., Hase T.P.A., Tanner B.K., Riveiro J.M. (2008). Artificial ferrimagnetic structure and thermal hysteresis in Gd0.47Co0.53/Co multilayers. Phys. Rev. B.

[26] Andrés J.P., Sacedón J.L., Colino J., Riveiro J.M. (2000). Interdiffusion up to the eutectic composition and vitrification in Gd/Co multilayers. J. Appl. Phys..

[27] Bertero G.A., Hufnagel T.C., Clemens B.M., Sinclair R. (1993). TEM analysis of Co-Gd and Co-Gd multilayer structures. J. Mater. Res..

[28] González J.A., Andrés J.P., Arranz M.A., López De La Torre M.A., Riveiro J.M. (2002). Interdiffusion and magnetic properties of Gd1-xCox/Co multilayers. J. Phys. Condens. Matter.

[29] González J.A., Andrés J.P., Arranz M.A., López de la Torre M.A., Riveiro J.M. (2002). Electrical resistivity and interdiffusion in Gd(1−x)Co(x)/Co multilayers. J. Appl. Phys..

[30] González J.A., Andrés J.P., López de la Torre M.A., Riveiro J.M., Hase T.P.A., Tanner B.K. (2003). X-ray study of the interdiffusion and interfacial structure in ferrimagnetic Gd_1−x_Co_x_/Co multilayers. J. Appl. Phys..

[31] Diény B. (1994). Dieny magnetism Giant magnetoresistance in spin-valve multilayers. J. Magn. Magn. Mater..

[32] Meiklejohn W.H., Bean C.P. (1956). New magnetic anisotropy. Phys. Rev..

[33] Nogués J., Schuller I.K. (1999). Exchange bias. J. Magn. Magn. Mater..

[34] Katti R.R. (2002). Current-in-plane pseudo-spin-valve device performance for giant magnetoresistive random access memory applications (invited). J. Appl. Phys..

[35] Samal D., Anil Kumar P.S. (2008). Giant magnetoresistance. Resonance.

[36] Bellouard C., George B., Marchal G., Maloufi N., Eugène J. (1997). Influence of the thickness of the CoFe layer on the negative spin-valve effect in CoFe/Ag/CoFeGd trilayers. J. Magn. Magn. Mater..

[37] Lai C.H., Lin C.C., Chen B.M., Shieh H.P.D., Chang C.R. (2001). Positive giant magnetoresistance in ferrimagnetic/Cu/ferrimagnetic films. J. Appl. Phys..

[38] Svalov A.V., Savin P.A., Kurlyandskaya G.V., Gutiérrez J., Barandiarán J.M., Vas V.O. (2002). Spin-Valve Structures with Co–Tb-Based Multilayers. IEEE Trans. Magn..

[39] Jiang X., Gao L., Sun J.Z., Parkin S.S.P. (2006). Temperature dependence of current-induced magnetization switching in spin valves with a ferrimagnetic CoGd free layer. Phys. Rev. Lett..

[40] Yang D.Z., You B., Zhang X.X., Gao T.R., Zhou S.M., Du J. (2006). Inverse giant magnetoresistance in FeCu Gd1-x Cox spin-valves. Phys. Rev. B-Condens. Matter Mater. Phys..

[41] Bai X.J., Du J., Zhang J., You B., Sun L., Zhang W., Hu A., Zhou S.M. (2008). Influence of the thickness of the FeCoGd layer on the magnetoresistance in FeCoGd-based spin valves and magnetic tunnel junctions. J. Phys. D Appl. Phys..

[42] Svalov A.V., Fernández A., Tejedor M., Kurlyandskaya G.V. (2007). The effect of the additional biasing on the switching process in pseudo spin-valve structure. Vacuum.

[43] Svalov A.V., Vas’kovskiy V.O., Orue I., Kurlyandskaya G.V. (2017). Tailoring of switching field in GdCo-based spin valves by inserting Co layer. J. Magn. Magn. Mater..

[44] Svalov A.V., Kurlyandskaya G.V., Vas’kovskiy V.O. (2016). Thermo-sensitive spin valve based on layered artificial ferrimagnet. Appl. Phys. Lett..

[45] Svalov A.V., Orue I., Kurlyandskaya G.V. (2018). Multi-step magnetization process of Gd-Co/Co/Cu/Co thermo-sensitive spin valves. Electronics.

[46] Svalov A.V., Stepanova E.A., Vas V.O., Kurlyandskaya G.V. (2019). Thermosensitive Spin Valve Based on an Artificial Ferrimagnet: Magnetization Process in a Wide Range of Fields. Phys. Solid State.

[47] Milyaev M., Naumova L., Chernyshova T., Proglyado V., Kamensky I., Krinitsina T., Ryabukhina M., Ustinov V. (2017). Magnetization reversal and inverted magnetoresistance of exchange-biased spin valves with a gadolinium layer. J. Appl. Phys..

[48] Romer S., Marioni M.A., Thorwarth K., Joshi N.R., Corticelli C.E., Hug H.J., Oezer S., Parlinska-Wojtan M., Rohrmann H. (2012). Temperature dependence of large exchange-bias in TbFe-Co/Pt. Appl. Phys. Lett..

[49] Schubert C., Hebler B., Schletter H., Liebig A., Daniel M., Abrudan R., Radu F., Albrecht M. (2013). Interfacial exchange coupling in Fe-Tb/[Co/Pt] heterostructures. Phys. Rev. B-Condens. Matter Mater. Phys..

[50] Heigl M., Vogler C., Mandru A.O., Zhao X., Hug H.J., Suess D., Albrecht M. (2020). Microscopic Origin of Magnetization Reversal in Nanoscale Exchange-Coupled Ferri/Ferromagnetic Bilayers: Implications for High Energy Density Permanent Magnets and Spintronic Devices. ACS Appl. Nano Mater..

[51] Hebler B., Reinhardt P., Katona G.L., Hellwig O., Albrecht M. (2017). Double exchange bias in ferrimagnetic heterostructures. Phys. Rev. B.

[52] Radu F., Abrudan R., Radu I., Schmitz D., Zabel H. (2012). Perpendicular exchange bias in ferrimagnetic spin valves. Nat. Commun..

[53] Radu I., Vahaplar K., Stamm C., Kachel T., Pontius N., Dürr H.A., Ostler T.A., Barker J., Evans R.F.L., Chantrell R.W. (2011). Transient ferromagnetic-like state mediating ultrafast reversal of antiferromagnetically coupled spins. Nature.

[54] Iusipova I.A. (2018). Analysis of the Switching Characteristics of MRAM Cells Based on Materials with Uniaxial Anisotropy. Semiconductors.

[55] Zhang W., Zhang D., Wong P.K.J., Yuan H., Jiang S., Van Der Laan G., Zhai Y., Lu Z. (2015). Selective Tuning of Gilbert Damping in Spin-Valve Trilayer by Insertion of Rare-Earth Nanolayers. ACS Appl. Mater. Interfaces.

[56] Chen Q., Ruan X., Yuan H., Zhou X., Kou Z., Huang Z., Xu Y., Zhai Y. (2020). Interlayer transmission of magnons in dynamic spin valve structures. Appl. Phys. Lett..

[57] Zabel H. (2009). Progress in spintronics. Superlattices Microstruct..

[58] Berger L. (1996). Emission of spin waves by a magnetic multilayer traversed by a current. Phys. Rev. B.

[59] Slonczewski J.C. (1996). Current-driven excitation of magnetic multilayers. J. Magn. Magn. Mater..

[60] Zhao W.S., Zhang Y., Devolder T., Klein J.O., Ravelosona D., Chappert C., Mazoyer P. (2012). Failure and reliability analysis of STT-MRAM. Microelectron. Reliab..

[61] Song C., Zhang R., Liao L., Zhou Y., Zhou X., Chen R., You Y., Pan F. (2020). Spin-orbit torques: Materials, mechanisms, performances, and potential applications. Prog. Mater. Sci..

[62] Hellman F., Hoffmann A., Tserkovnyak Y., Beach G.S.D., Fullerton E.E., Leighton C., MacDonald A.H., Ralph D.C., Arena D.A., Dürr H.A. (2017). Interface-Induced Phenomena in Magnetism. Rev. Mod. Phys..

[63] Manchon A., Koo H.C., Nitta J., Frolov S.M., Duine R.A. (2015). New perspectives for Rashba spin-orbit coupling. Nat. Mater..

[64] Ramaswamy R., Lee J.M., Cai K., Yang H. (2018). Recent advances in spin-orbit torques: Moving towards device applications. Appl. Phys. Rev..

[65] Yang T., Matsumoto T., Yamane H., Kamiko M., Yamamoto R. (1999). Perpendicular magnetic anisotropy and magneto-optical properties of (Co-Tb)/Pd multilayers. J. Magn. Magn. Mater..

[66] Huang K.F., Wang D.S., Lin H.H., Lai C.H. (2015). Engineering spin-orbit torque in Co/Pt multilayers with perpendicular magnetic anisotropy. Appl. Phys. Lett..

[67] Jamali M., Narayanapillai K., Qiu X., Loong L.M., Manchon A., Yang H. (2013). Spin-orbit torques in Co/Pd multilayer nanowires. Phys. Rev. Lett..

[68] Ueda K., Mann M., Pai C.F., Tan A.J., Beach G.S.D. (2016). Spin-orbit torques in Ta/TbxCo100-xferrimagnetic alloy films with bulk perpendicular magnetic anisotropy. Appl. Phys. Lett..

[69] Ueda K., Tan A.J., Beach G.S.D. (2018). Effect of annealing on magnetic properties in ferrimagnetic GdCo alloy films with bulk perpendicular magnetic anisotropy. AIP Adv..

[70] Subbotin I.A., Pashaev E.M., Vasiliev A.L., Chesnokov Y.M., Prutskov G.V., Kravtsov E.A., Makarova M.V., Proglyado V.V., Ustinov V.V. (2019). The influence of microstructure on perpendicular magnetic anisotropy in Co/Dy periodic multilayer systems. Phys. B Condens. Matter.

[71] Chen X., Wang Y.J., Liang B.Q., Tang Y.J., Zhao H.W., Xiao J.Q. (2000). Perpendicular anisotropy in the amorphous TbCo/Si multilayers. J. Appl. Phys..

[72] Mishra R., Yu J., Qiu X., Motapothula M., Venkatesan T., Yang H. (2017). Anomalous Current-Induced Spin Torques in Ferrimagnets near Compensation. Phys. Rev. Lett..

[73] Roschewsky N., Matsumura T., Cheema S., Hellman F., Kato T., Iwata S., Salahuddin S. (2016). Spin-orbit torques in ferrimagnetic GdFeCo alloys. Appl. Phys. Lett..

[74] Roschewsky N., Lambert C.H., Salahuddin S. (2017). Spin-orbit torque switching of ultralarge-thickness ferrimagnetic GdFeCo. Phys. Rev. B.

[75] Finley J., Liu L. (2016). Spin-Orbit-Torque Efficiency in Compensated Ferrimagnetic Cobalt-Terbium Alloys. Phys. Rev. Appl..

[76] Han J., Richardella A., Siddiqui S.A., Finley J., Samarth N., Liu L. (2017). Roomerature Spin-Orbit Torque Switching Induced by a Topological Insulator. Phys. Rev. Lett..

[77] Wu H., Xu Y., Deng P., Pan Q., Razavi S.A., Wong K., Huang L., Dai B., Shao Q., Yu G. (2019). Spin-Orbit Torque Switching of a Nearly Compensated Ferrimagnet by Topological Surface States. Adv. Mater..

[78] Treutler C.P.O. (2001). Magnetic sensors for automotive applications. Sens. Actuators A Phys..

[79] Luo Z., Xu Y., Yang Y., Wu Y. (2018). Magnetic angular position sensor enabled by spin-orbit torque. Appl. Phys. Lett..

[80] Xie H., Chen X., Luo Z., Wu Y. (2021). Spin Torque Gate Magnetic Field Sensor. Phys. Rev. Appl..

[81] Berger L. (1984). Exchange interaction between ferromagnetic domain wall and electric current in very thin metallic films. J. Appl. Phys..

[82] Freitas P.P., Berger L. (1985). Observation of s-d exchange force between domain walls and electric current in very thin Permalloy films. J. Appl. Phys..

[83] Bang D., Van Thach P., Awano H. (2018). Current-induced domain wall motion in antiferromagnetically coupled structures: Fundamentals and applications. J. Sci. Adv. Mater. Devices.

[84] Bang D., Yu J., Qiu X., Wang Y., Awano H., Manchon A., Yang H. (2016). Enhancement of spin Hall effect induced torques for current-driven magnetic domain wall motion: Inner interface effect. Phys. Rev. B.

[85] Kim K.J., Kim S.K., Hirata Y., Oh S.H., Tono T., Kim D.H., Okuno T., Ham W.S., Kim S., Go G. (2017). Fast domain wall motion in the vicinity of the angular momentum compensation temperature of ferrimagnets. Nat. Mater..

[86] Kurokawa Y., Kawamoto M., Awano H. (2016). Current-induced domain wall motion attributed to spin Hall effect and Dzyaloshinsky-Moriya interaction in Pt/GdFeCo (100 nm) magnetic wire. Jpn. J. Appl. Phys..

[87] Kim K.J., Lee J.C., Choe S.B., Shin K.H. (2008). Joule heating in ferromagnetic nanowires: Prediction and observation. Appl. Phys. Lett..

[88] Parkin S.S.P., Hayashi M., Thomas L. (2008). Magnetic Domain-Wall Racetrack Memory. Science.

[89] Yang S.H., Ryu K.S., Parkin S. (2015). Domain-wall velocities of up to 750 m s-1driven by exchange-coupling torque in synthetic antiferromagnets. Nat. Nanotechnol..

[90] Parkin S., Yang S.H. (2015). Memory on the racetrack. Nat. Nanotechnol..

[91] Bläsing R., Ma T., Yang S.H., Garg C., Dejene F.K., N’Diaye A.T., Chen G., Liu K., Parkin S.S.P. (2018). Exchange coupling torque in ferrimagnetic Co/Gd bilayer maximized near angular momentum compensation temperature. Nat. Commun..

[92] Ueda K., Mann M., De Brouwer P.W.P., Bono D., Beach G.S.D. (2017). Temperature dependence of spin-orbit torques across the magnetic compensation point in a ferrimagnetic TbCo alloy film. Phys. Rev. B.

[93] Stanciu C.D., Kimel A.V., Hansteen F., Tsukamoto A., Itoh A., Kirilyuk A., Rasing T. (2006). Ultrafast spin dynamics across compensation points in ferrimagnetic GdFeCo: The role of angular momentum compensation. Phys. Rev. B Condens. Matter Mater. Phys..

[94] Binder M., Weber A., Mosendz O., Woltersdorf G., Izquierdo M., Neudecker I., Dahn J.R., Hatchard T.D., Thiele J.U., Back C.H. (2006). Magnetization dynamics of the ferrimagnet CoGd near the compensation of magnetization and angular momentum. Phys. Rev. B Condens. Matter Mater. Phys..

[95] Hirata Y., Kim D.H., Okuno T., Nishimura T., Kim D.Y., Futakawa Y., Yoshikawa H., Tsukamoto A., Kim K.J., Choe S.B. (2018). Correlation between compensation temperatures of magnetization and angular momentum in GdFeCo ferrimagnets. Phys. Rev. B.

[96] Sampaio J., Grollier J., Metaxas P.J. (2015). Domain Wall Motion in Nanostructures.

[97] Allwood D.A., Xiong G., Faulkner C.C., Atkinson D., Petit D., Cowburn R.P. (2005). Magnetic domain-wall logic. Science.

[98] Diegel M., Mattheis R., Halder E. (2007). Multiturn counter using movement and storage of 180° magnetic domain walls. Sens. Lett..

[99] Diegel M., Glathe S., Mattheis R., Scherzinger M., Haider E. (2009). A new four bit magnetic domain wall based multiturn counter. IEEE Trans. Magn..

[100] Stanciu C.D., Hansteen F., Kimel A.V., Kirilyuk A., Tsukamoto A., Itoh A., Rasing T. (2007). All-optical magnetic recording with circularly polarized light. Phys. Rev. Lett..

[101] Kaka S., Russek S.E. (2002). Precessional switching of submicrometer spin valves. Appl. Phys. Lett..

[102] Back C.H., Allenspach R., Weber W., Parkin S.S.P., Weller D., Garwin E.L., Siegmann H.C. (1999). Minimum field strength in precessional magnetization reversal. Science.

[103] Tudosa I., Stamm C., Kashuba A.B., King F., Siegmann H.C., Stöhr J., Ju G., Lu B., Weeler D. (2004). The ultimate speed of magnetic switching in granular recording media. Nature.

[104] Beaurepaire E., Merle J.C., Daunois A., Bigot J.Y. (1996). Ultrafast spin dynamics in ferromagnetic nickel. Phys. Rev. Lett..

[105] Ostler T.A., Barker J., Evans R.F.L., Chantrell R.W., Atxitia U., Chubykalo-Fesenko O., El Moussaoui S., Le Guyader L., Mengotti E., Heyderman L.J. (2012). Ultrafast heating as a sufficient stimulus for magnetization reversal in a ferrimagnet. Nat. Commun..

[106] Hassdenteufel A., Hebler B., Schubert C., Liebig A., Teich M., Helm M., Aeschlimann M., Albrecht M., Bratschitsch R. (2013). Thermally assisted all-optical helicity dependent magnetic switching in amorphous Fe100-xTbx alloy films. Adv. Mater..

[107] Alebrand S., Gottwald M., Hehn M., Steil D., Cinchetti M., Lacour D., Fullerton E.E., Aeschlimann M., Mangin S. (2012). Light-induced magnetization reversal of high-anisotropy TbCo alloy films. Appl. Phys. Lett..

[108] Alebrand S., Bierbrauer U., Hehn M., Gottwald M., Schmitt O., Steil D., Fullerton E.E., Mangin S., Cinchetti M., Aeschlimann M. (2014). Subpicosecond magnetization dynamics in TbCo alloys. Phys. Rev. B Condens. Matter Mater. Phys..

[109] Ciuciulkaite A., Mishra K., Moro M.V., Chioar I.A., Rowan-Robinson R.M., Parchenko S., Kleibert A., Lindgren B., Andersson G., Davies C.S. (2020). Magnetic and all-optical switching properties of amorphous TbxCo100-x alloys. Phys. Rev. Mater..

[110] Lalieu M.L.M., Peeters M.J.G., Haenen S.R.R., Lavrijsen R., Koopmans B. (2017). Deterministic all-optical switching of synthetic ferrimagnets using single femtosecond laser pulses. Phys. Rev. B.

[111] Mangin S., Gottwald M., Lambert C.H., Steil D., Uhlíř V., Pang L., Hehn M., Alebrand S., Cinchetti M., Malinowski G. (2014). Engineered materials for all-optical helicity-dependent magnetic switching. Nat. Mater..

[112] Vahaplar K., Kalashnikova A.M., Kimel A.V., Gerlach S., Hinzke D., Nowak U., Chantrell R., Tsukamoto A., Itoh A., Kirilyuk A. (2012). All-optical magnetization reversal by circularly polarized laser pulses: Experiment and multiscale modeling. Phys. Rev. B Condens. Matter Mater. Phys..

[113] Lambert C.H., Mangin S., Varaprasad B.S.D.C.S., Takahashi Y.K., Hehn M., Cinchetti M., Malinowski G., Hono K., Fainman Y., Aeschlimann M. (2014). All-optical control of ferromagnetic thin films and nanostructures. Science.

[114] El Hadri M.S., Pirro P., Lambert C.H., Bergeard N., Petit-Watelot S., Hehn M., Malinowski G., Montaigne F., Quessab Y., Medapalli R. (2016). Electrical characterization of all-optical helicity-dependent switching in ferromagnetic Hall crosses. Appl. Phys. Lett..

[115] Sander D., Valenzuela S.O., Makarov D., Marrows C.H., Fullerton E.E., Fischer P., Mccord J., Vavassori P., Mangin S., Pirro P. (2017). The 2017 Magnetism Roadmap. J. Phys. D Appl. Phys.

[116] El Hadri M.S., Pirro P., Lambert C.H., Petit-Watelot S., Quessab Y., Hehn M., Montaigne F., Malinowski G., Mangin S. (2016). Two types of all-optical magnetization switching mechanisms using femtosecond laser pulses. Phys. Rev. B.

[117] El Hadri M.S., Hehn M., Malinowski G., Mangin S. (2017). Materials and devices for all-optical helicity-dependent switching. J. Phys. D Appl. Phys..

[118] Gorchon J., Wilson R.B., Yang Y., Pattabi A., Chen J.Y., He L., Wang J.P., Li M., Bokor J. (2016). Role of electron and phonon temperatures in the helicity-independent all-optical switching of GdFeCo. Phys. Rev. B.

[119] Davies C.S., Janssen T., Mentink J.H., Tsukamoto A., Kimel A.V., Van Der Meer A.F.G., Stupakiewicz A., Kirilyuk A. (2020). Pathways for Single-Shot All-Optical Switching of Magnetization in Ferrimagnets. Phys. Rev. Appl..

[120] Liu T.M., Wang T., Reid A.H., Savoini M., Wu X., Koene B., Granitzka P., Graves C.E., Higley D.J., Chen Z. (2015). Nanoscale Confinement of All-Optical Magnetic Switching in TbFeCo - Competition with Nanoscale Heterogeneity. Nano Lett..

